# Participatory design in the development of an early therapy intervention for perinatal stroke

**DOI:** 10.1186/s12887-017-0797-9

**Published:** 2017-01-23

**Authors:** Anna Purna Basu, Janice Elizabeth Pearse, Jessica Baggaley, Rose Mary Watson, Tim Rapley

**Affiliations:** 1 Institute of Neuroscience, Newcastle University, Newcastle upon Tyne, NE1 7RU UK; 2 Department of Paediatric Neurology, Newcastle upon Tyne Hospitals NHS Foundation Trust, Newcastle upon Tyne, NE7 7DN UK; 3 Therapy Services, Newcastle upon Tyne Hospitals NHS Foundation Trust, Newcastle upon Tyne, NE7 7DN UK; 4 Medical Sciences Graduate School, Newcastle University, Newcastle upon Tyne, NE1 7RU UK; 5 Institute of Health and Society, Newcastle University, Newcastle upon Tyne, NE2 4AX UK

**Keywords:** Intervention development, Perinatal stroke, Early intervention, Therapy, Motor system, Normalisation process theory, Participatory design, Unilateral cerebral palsy, Hemiparesis, Infant

## Abstract

**Background:**

Perinatal stroke is the leading cause of unilateral (hemiparetic) cerebral palsy, with life-long personal, social and financial consequences. Translational research findings indicate that early therapy intervention has the potential for significant improvements in long-term outcome in terms of motor function. By involving families and health professionals in the development and design stage, we aimed to produce a therapy intervention which they would engage with.

**Methods:**

Nine parents of children with hemiparesis and fourteen health professionals involved in the care of infants with perinatal stroke took part in peer review and focus groups to discuss evolving therapy materials, with revisions made iteratively. The materials and approach were also discussed at a meeting of the London Child Stroke Research Reference Group. Focus group data were coded using Normalisation Process Theory constructs to explore potential barriers and facilitators to routine uptake of the intervention.

**Results:**

We developed the Early Therapy in Perinatal Stroke (eTIPS) program - a parent-delivered, home-based complex intervention addressing a current gap in practice for infants in the first 6 months of life after unilateral perinatal stroke and with the aim of improving motor outcome. Parents and health professionals saw the intervention as different from usual practice, and valuable (high coherence). They were keen to engage (high cognitive participation). They considered the tasks for parents to be achievable (high collective action). They demonstrated trust in the approach and felt that parents would undertake the recommended activities (high collective action). They saw the approach as flexible and adaptable (high reflexive monitoring). Following suggestions made, we added a section on involving the extended family, and obtained funding for a website and videos to supplement written materials.

**Conclusions:**

Focus groups with parents and health professionals provided meaningful feedback to iteratively improve the intervention materials prior to embarking on a pilot study. The intervention has a high potential to normalize and become a routine part of parents’ interactions with their child following unilateral perinatal stroke.

## Background

Perinatal stroke is due to an interrupted blood supply to part of the brain before birth or age <28 days [1–4]. The condition affects up to 1/2300 term [1, 5] and 7/1000 preterm infants [1, 6], 60% of whom acquire neurological deficits as a consequence [7–9]. Perinatal stroke is a common cause of unilateral cerebral palsy [3, 6, 7, 10–13], with long-term adverse effects on activities of daily living [14], quality of life and self-esteem [15]. The aetiology of perinatal stroke is multifactorial [9] and incompletely understood [16], limiting preventative options. Early detection also remains challenging, as presentation is non-specific, with seizures [17], lethargy and/or poor feeding [18]; around 40% (“presumed perinatal strokes” [10, 11, 13, 19]), are first detected many months after birth, often with emerging movement difficulties [20–22]. Currently the options for immediate treatment of perinatal stroke remain few, though this is an area of research interest [23]. Effective early intervention for perinatal stroke has the potential for lifelong benefits [24].

A case notes review covering a 10 year period [25], a national survey of therapist management of perinatal stroke undertaken by our group (under submission) and reviews of the literature [23, 26] all indicated the lack of any standardised therapy approach for perinatal stroke in the first 6 months of life, though there is current research into modified constraint [27] and action-observation [28, 29] approaches starting from around 3 months of age in infants with emerging motor asymmetries regardless of aetiology. Despite the obvious challenges in delivery, the basic science literature from neurophysiological [30, 31], animal [32] and behavioural [33] studies suggests that for infants with predominantly unilateral stroke who are at risk of unilateral cerebral palsy, early intervention could improve long-term motor outcome. The rationale for this approach is provided below.

### Activity-dependent competition shapes corticospinal tract projections

The corticospinal tract is a major descending pathway from the brain to the spinal cord controlling skilled voluntary movement. Neurophysiological studies using transcranial magnetic stimulation demonstrate marked activity-dependent developmental and post-lesional corticospinal tract changes in the first months of life [34–36]. At term, the corticospinal tract projects bilaterally from each hemisphere to the spinal cord. An activity-dependent process drives a gradual progression to a predominantly crossed corticospinal tract projection in typically developing infants [37]. Perinatal stroke perturbs the outcome of this process to a variable extent: the best functional outcome is seen in patients who retain a crossed corticospinal tract contribution from the affected hemisphere [34]. In contrast, in patients with a poor motor outcome there is a gradual loss of crossed projections from the affected hemisphere with abnormal retention of uncrossed projections from the unaffected hemisphere [37, 38]. Animal models produce similar results and confirm the activity-dependent nature of the process [39]. In addition, animal models have shown that the loss of corticospinal tract fibres following stroke leads to sprouting of proprioceptive afferents even in adults, leading to hyperreflexia [40]. In infant animal models, it is clear that damage to the corticospinal tract during development has secondary effects on spinal cord interneuron populations [41]. The corticospinal tract also shapes development of subcortical motor systems through activity-dependent mechanisms: unilateral CST damage adversely affects the descending rubrospinal tract pathways [42]. These developmental corticospinal tract and spinal cord changes offer unique opportunities for intervention in infants with perinatal stroke in a dynamic, activity-dependent system with no parallel in adults.

### Promoting activity in the potentially affected limb improves outcome

Animal studies indicate that the consequences of perinatal stroke can be mitigated by promoting activity of the potentially affected side. In a study undertaken in kittens, muscimol was used to silence the motor cortex unilaterally, leading to an abnormal pattern of corticospinal tract projections and adverse consequences for motor function. By using electrical stimulation of the inactivated corticospinal tract fibres, the normal pattern of corticospinal tract projection was partially restored and motor function also improved [35]. Subsequently it was shown that early therapy intervention (constraint plus training on a reaching task), rather than invasive electrical stimulation of the corticospinal tract, led to similar improvements in this animal model [43]. However, constraint is problematic as an immediate intervention following neonatal stroke because of the potential for harm [44–47]. Instead, early environmental manipulation to promote activity of the potentially affected side is proposed [48].

### Environmental manipulations can influence activity from an early age

From birth, infants will demonstrate early “pre-reaching” movements preferentially with the arm nearest a toy presented to one side [49]. The play environment can be manipulated to encourage activity of the potentially affected side. This principle can be extended to a pervasive intervention affecting the carer-based [50], play-based and physical environment around the baby, delivered in the home with therapist support [50–52].

The immaturity of the infant motor system at birth means that in the first months after unilateral perinatal stroke, no motor asymmetries may be detectable [13, 53], though general movements assessments may show unilaterally absent fidgety movements at around 3 months of age [54]. This adds to the difficulties of introducing and explaining a therapy approach aiming to promote activity of the potentially affected side of the body during the “silent period” [13, 53] when lateralised motor signs are absent, even where imaging findings are strongly predictive of an outcome of unilateral cerebral palsy [55–58]. It was essential to involve stakeholders from an early stage in developing an early therapy intervention approach which would be acceptable and deliverable in this context.

We aimed to develop a manualised, parent-delivered, home-based early therapy intervention for the first 6 months of life, to improve motor function in infants with predominantly unilateral perinatal stroke. Medical Research Council (MRC) guidance on complex interventions was followed [59].

## Methods

### Rationale for initial content

Based on the literature discussed above, we chose an approach suitable for infants with predominantly unilateral perinatal stroke including haemorrhagic parenchymal infarcts, aiming to promote activity of the potentially affected side of the body during a time period of active central nervous system plasticity and ongoing corticospinal tract wiring. We named the approach “Early Therapy in Perinatal Stroke”, abbreviated to eTIPS.

We chose an input in the first 6 months of life because this was identified as being the period during which the greatest changes in corticospinal tract wiring occurred postnatally [30], as well as a period during which there was no consensus regarding the approach to assessment and intervention [25]. In addition, infants in this age range are relatively immobile so lateralisation of the environment around the infant remains possible.

The initial content of the therapy was based on identifying aspects of everyday life for the infant for which a lateralised approach could be provided – i.e., increasing sensory input and opportunities for movement of the potentially affected side of the body. A parent-delivered therapy intervention seemed the most appropriate option because in the first months of life the infant is usually looked after by a small number of close family members. A therapy designed to utilise that input within the context of the carer/child relationship could be delivered in a far greater dose than that achievable through therapist input alone. We were mindful of the potential difficulty parents might have in being cast in the role of therapist, and aimed to make the approach and manual as accessible, pervasive and play-based as possible.

Of major importance was the issue of achieving pervasiveness of the approach so that the therapy dose would be high, whilst minimising the burden to parents. Pervasive but relatively minor changes to everyday activities were felt to be more deliverable than therapy blocks based on infant lifestyle and attention span considerations. In addition, a lateralised approach can be applied during many aspects of the infant’s daily routines, potentially providing a large therapy dose through a bioecological approach [60].

Logically, the most appropriate setting for a parent-delivered, pervasive therapy intervention is in the home, centred round the microsystem of the infant. This approach has been used for other therapy interventions in children with cerebral palsy [52, 61, 62], and our group has experience with developing and overseeing home-based parent-delivered therapy approaches in children with unilateral cerebral palsy [63].

**Fig. 1:**
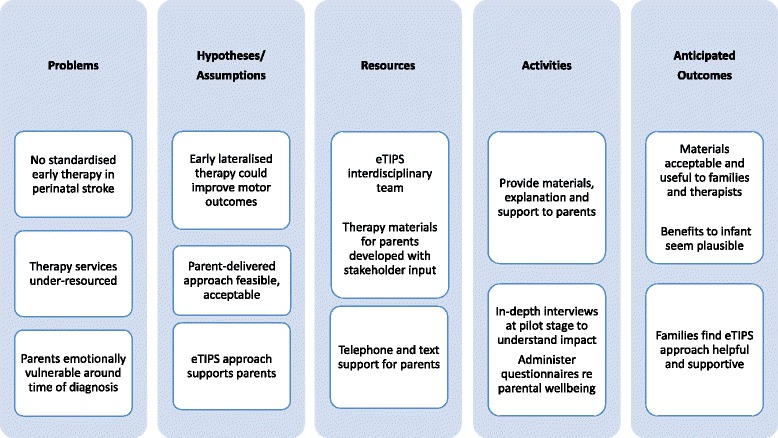
Logic Model

Another important aspect was to keep the tone of the manual and materials positive and to make sure the activities were enjoyable for parents, other members of the support/carer network and for the infants themselves. An experienced paediatric occupational therapist was key to this stage of development and design. A logic model, or conceptual framework was developed for the approach [64] (Fig. 1).

### Participatory design process

**Fig. 2:**
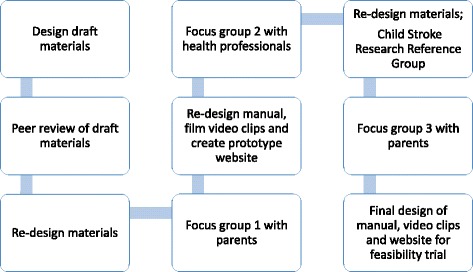
Outline of participatory design process

Participatory design, also known as co-operative design [65], is a process in which the proposed end-users of a product are actively involved in the design of the product. An iterative approach is often taken, with users evaluating the product, modifications made and re-evaluation undertaken over several cycles. The aim is to improve the quality and value of the final product to end-users. Focus groups are a valuable approach to use, enabling demonstrations of prototypes and the provision of opportunities for discussion and feedback. We chose to use a participatory design process, discussing the materials and concepts with key stakeholders to increase the likelihood that the approach would be understood, adopted and normalised [66]. Following an initial peer review group meeting, three focus groups were held (two groups with parents of children with unilateral cerebral palsy secondary to perinatal stroke, and one with therapists and other healthcare professionals). Revisions to the materials were made iteratively in line with feedback from the groups as shown in Fig. 2.

### Setting

Peer review and focus groups were undertaken in Newcastle upon Tyne. Feedback was also obtained from the London Child Stroke Research Reference Group (Evelina Hospital/Stroke Association).

### Participants

A stratified purposive sampling approach was used, focussing on key groups of stakeholders [67]. We aimed for 6–10 parents or health professionals per group, representing an appropriate size for a focus group to confirm/develop prototype design [68]. A total of nine parents of children aged 3–18 years with unilateral cerebral palsy due to perinatal stroke took part in two focus groups. We also held one peer review group and one focus group with a total of sixteen health care professionals including community and hospital paediatric physiotherapists and occupational therapists, neonatal physiotherapists, a play specialist and a paediatric neurodisability consultant. Recruitment was through a local parent support group (HemiHelp), Newcastle upon Tyne Hospitals NHS Foundation Trust, and (for therapists), through regional therapist networks. Adequate English language skills to participate in the focus groups were a requirement for inclusion. We planned to exclude parents of affected children under the age of 4 years to avoid undue distress to families in whom the diagnosis might still be evolving. However, two parents from the local support group with children aged 3 years were extremely keen to participate and felt they could do so safely, so were included. Fully informed written consent was obtained prior to participation, and ethical approval was obtained (North of Scotland Research Ethics Committee 2, ref. 14/NS/1027).

Further feedback was obtained through presentation of the proposed materials to a small established group of parents of children with stroke (onset at any age) at the London Child Stroke Research Reference Group meeting at the Evelina Hospital, organised by a senior consultant occupational therapist and a Child Stroke Project Manager for the Stroke Association). This occurred prior to the final parents’ focus group.

### Focus groups

A topic guide was developed for the focus groups and ground rules were established at the start. The main areas of discussion were around the rationale for, and nature of the planned intervention. Draft materials including the manual and mock-up video clips illustrating aspects of the therapy approach were reviewed interactively. We asked whether any components of the proposed materials should be changed or removed, and if new information should be added. We were interested in potential barriers to uptake of the intervention, and how these could be overcome. We also wanted to know how parents and therapists felt the approach would fit in with their role. We also remained open to broader suggestions and thoughts about the approach.

The peer review and focus groups were audio recorded and transcribed verbatim, then anonymised.

Anonymised transcripts formed the data for formal analysis, theoretically informed by Normalisation Process Theory (NPT) [69]. NPT provides a theoretical framework for considering barriers and facilitators to “normalisation”, i.e., of widespread implementation and incorporation into routine practice, of complex interventions. The approach fits in well with the ethos of the participatory design process, which has very similar aims. By using the NPT framework at the stage of intervention development, potential barriers to uptake can be identified and addressed early [66]. We therefore chose this framework to give us an understanding of how well the eTIPS approach might become embedded into routine practice from the point of view of parents and therapists, and where there might be barriers to overcome. The four main NPT constructs are described as coherence, cognitive participation, collective action and reflexive monitoring. The first construct, coherence, covers the extent to which people can make sense of the intervention and its purpose, and understand how it differs from other interventions. The second construct, cognitive participation, describes the degree of engagement with the intervention and willingness to commit to it over time. The third construct, collective action, covers the work required to adopt the new process, i.e., the necessary behaviour changes and actions as well as access to skills and resources. The fourth construct, reflexive monitoring, describes the ongoing appraisal of the intervention and any adjustments necessary over time to ensure sustainability and integration into practice. At the stage of intervention development, the first three constructs are of most relevance, whereas reflexive monitoring is more relevant to subsequent evaluative and implementation stages.

All analyses were conducted according to the standard procedures of rigorous qualitative analysis [70]. We used procedures from first-generation grounded theory (coding, constant comparison, memoing) [71], from analytic induction (deviant case analysis) [72] and from constructionist grounded theory (mapping) [73]. We undertook independent coding and cross checking and a proportion of data was analysed collectively in data clinics where the research team share and exchange interpretations of key issues emerging from the data. After group discussion, amendments were made to the materials as a result of feedback from the focus groups.

## Results

**Fig. 3:**
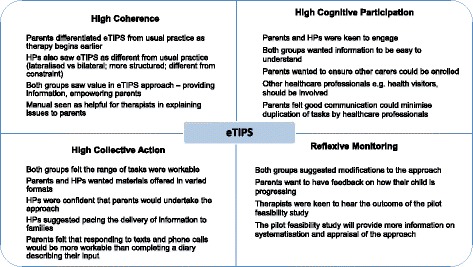
Focus group outputs mapped to NPT

Figure 3 shows the focus group outputs mapped to the four NPT constructs and showing high levels of coherence, cognitive participation and collective action. Rather than encountering potential barriers to implementation, we were provided with many helpful suggestions for optimisation of the approach. More information about the fourth construct (reflexive monitoring, i.e., ongoing appraisal and adjustments to practice) will emerge over time during the pilot feasibility study which we are currently undertaking. Focus group participants (parents and healthcare professionals) were vocal both in their support for the approach and in their range of suggestions for improvements. There were discussions about how infants would be identified (namely, through cranial imaging following presentation with symptomatic perinatal stroke, or as an incidental finding on routine cranial imaging of preterm infants). Parents described varying experiences in terms of promptness or otherwise of diagnosis following initial presentation: they were aware that some infants would not be diagnosed (with presumed perinatal stroke) until after the first 6 months of life. Parents and healthcare professionals talked about the varying terminology used at diagnosis, and the perceived uncertainty around outcome. Therapists could see the challenges in explaining the rationale for a lateralised therapy in infants who had not yet developed lateralised motor signs, even where these were expected to emerge based on imaging findings. In the following quote, a therapist is describing a potential scenario in which a parent of a young infant with a left hemispheric perinatal stroke, who is at high risk of developing a right hemiparesis but has not yet done so, might struggle to understand why a therapist is focussed on encouraging facilitation of active movement of the right side of the body:

But it’s actually introducing stuff when the family haven’t got the grasp that there is the problem. So for them to go in and say, “Right, right side, right side, right side” is sometimes the overwhelming bit. – Therapist, Peer review group.

Comments on the materials were focussed on optimisation; essentially, participants were confident that the eTIPS pervasive approach was achievable, with smooth embedding:

I think it’s good because it, it sort of normalises the whole thing. … these are the things you can do with your baby that you do anyway but just add into that just a little bit extra. … So I think for parents it’s quite user friendly. - Children’s Community Occupational Therapist, Focus group 2

My little girl, she was diagnosed at birth, er, so we, this would’ve been great for us, really, really helpful. – Mother, Focus group 3

Parents mentioned the strong sense of guilt and self-blame experienced after a diagnosis of perinatal stroke – a recognised problem [74, 75]:

… you blame yourself and then you, you think that everyone else is blaming you as well. – Mother, Focus group 1

They wanted the materials to include a message that parents are not to blame for this condition, and also some information on what to say to other family members including siblings. The need for more psychological support for parents was expressed:

… in my opinion, as soon as your child is diagnosed with a disability you should be referred for counselling. As for my personal opinion, and I, I’ve never, this is the hardest thing I’ve ever had to deal with in my life. -Mother, Focus group 3

**Fig. 4:**
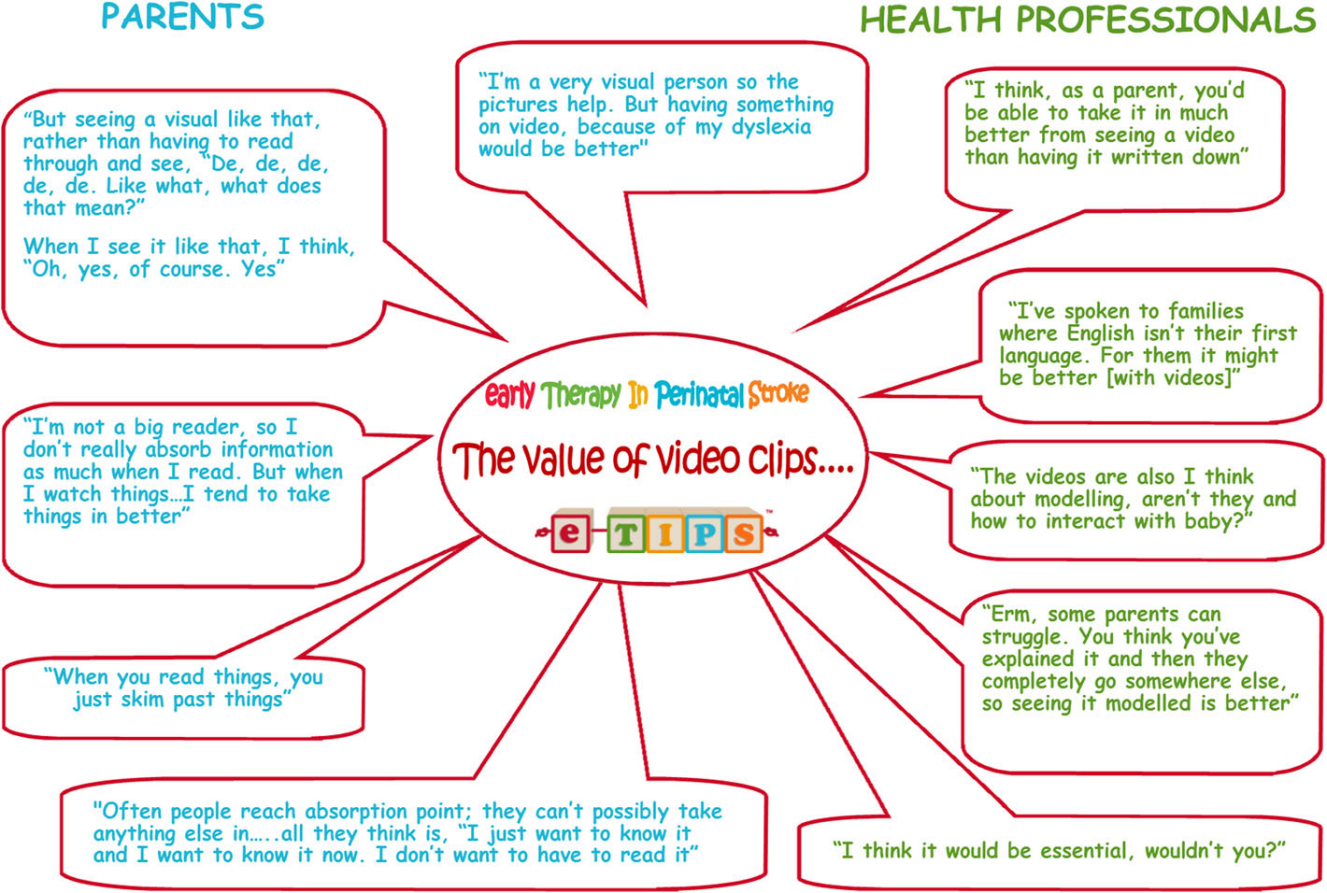
Quotes advocating for the inclusion of video clips in the materials for parents

Parents and professionals were keen to have materials available in multiple formats, particularly with videos and photographs to illustrate certain aspects of the approach. Parents were also keen to have the materials available through a website. The rationale for the multiple formats was based on variations in parental preference and learning styles. Parents also wanted the materials to be as simple and accessible as possible, given the high levels of parental exhaustion and sleep deprivation in the first months after childbirth. Quotes on the value of incorporating video clips demonstrating aspects of the approach are shown in Fig. 4. The London Child Stroke Research Reference Group suggested contact through phone calls and text reminders as a valuable method of keeping parents on track and obtaining feedback; they advised against requiring regular therapy diary completion by parents.

Therapists were concerned that parents might be overwhelmed if given all the materials at once. They suggested breaking down the education process into manageable chunks. Therapists were also concerned that the infant might be overwhelmed and suggested ways of pacing the activities and recognising signals or cues from the infant suggesting that a break was needed. Interestingly, parents expressed experiencing a lack of information provision and wanted as much information as possible including details of other agencies which might be involved and what to do and expect “next”, i.e., after the eTIPS intervention period was complete:

In our first 6 months, it’s – we didn’t get hardly any information at all apart from the health visitor. – Father, Focus group 1

A, a huge manual sometimes just overwhelms them and they’ll put it in the, put them in the drawer. – Therapist, peer review group

Therapists were able to distinguish between the eTIPS approach and their usual approach to infants after perinatal stroke before any lateralised signs are present. They discussed the regional variability in therapy services provision and recognised that some families would be able to access more therapist support than others – one therapist commented on a 12 week wait for physiotherapy services in one area even for infants considered to be a high priority. They saw the eTIPS materials as potentially beneficial in that they would facilitate explanation to families and could be used flexibly during home visits:

That’s what I think is quite nice, is that I could actually go, even if the baby was asleep. … I could still go, and go through some of this with, with the parent. – Therapist, Peer review group.

There was some discussion as to which healthcare professionals would be best placed to provide support for the eTIPS approach long-term: community physiotherapists or health visitors were suggested. This tied in with expressed parental wishes to avoid any duplication of assessments through research and clinical teams.

Table 1 summarises the key changes made as a result of the participatory design process. Information from the focus groups (including Fig. 4) was used to support funding applications for input from a professional film-maker, a professional photographer and a website designer. This was important given the request for provision of information in multiple formats, and to make the materials as visually appealing as possible. Photoshoots and films were planned and directed by members of the research team, to illustrate key aspects of the approach. Given the potential for right-left confusion in people from all educational backgrounds [76], we in fact developed two separate sets of materials – one for infants at risk of developing right hemiparesis and one for those at risk of left hemiparesis. Professional photoshoots and videos were shot separately for each. The availability of photos, videos and instructions tailored to the relevant side of the body makes it as easy as possible for parents to reproduce the required environmental and behavioural adaptations.

**Table 1:** Changes made to materials as a result of feedback

Changes		Parents	Healthcare professionals
Terminology	Simplification of text	y	y
	Avoidance of emotionally “loaded” words	y	y
Parental emotions	“It’s not your fault”	y	
Parental expectations	“What Next” section to cover the period after the intervention ends	y	
Information for parents	Safety (sleeping arrangements: “Back to Sleep” campaign)	y	y
	Describe other agencies which may be involved	y	
	Explanation for siblings	y	y
Improving fidelity	Text reminders	y	
Presentation	Videos and website	y	y
	Reduce content on some pages		y
General approach	Emphasize play-based, fun aspects		y
Consider infant’s attention, learning and infant cues	Calm environment; incorporate repetition; “one toy at a time”; reading infant cues/signals		y
Consider parental attention and learning	Parents may need information broken down in chunks		y

To accommodate the varying opinions about volume and pacing of information, the video clips were made to be bite-sized and the manual was divided into discrete sections, with the addition of a main message section and a summary leaflet or “miniguide”. Summary “mind maps” and checklists for parents were included to help them prepare mentally and practically for adoption of the eTIPS approach. We also included a ribbon in the pack for families, which we could tie to one side of the pram, chair or crib to help with right-left orientation. Guidance on reading and responding to infant cues was included in the manual. We incorporated emoticon-style stickers into the manuals so that families could easily mark sections for discussion during the visits, with the aim of making parental feedback as straightforward as possible. Many suggestions for simplification of language and layout were adopted, making the final materials (which were also proofread by several lay readers) very straightforward and accessible. Specifically, each page of the manual contains very short passages of text, in large font, broken up with illustrative photographs.

It was not possible to incorporate formal psychologist input into the eTIPS materials because of resource limitations. However, clinicians need to be aware of this largely unmet need and to have a lower threshold for referral to existing services.

**Fig. 5:**
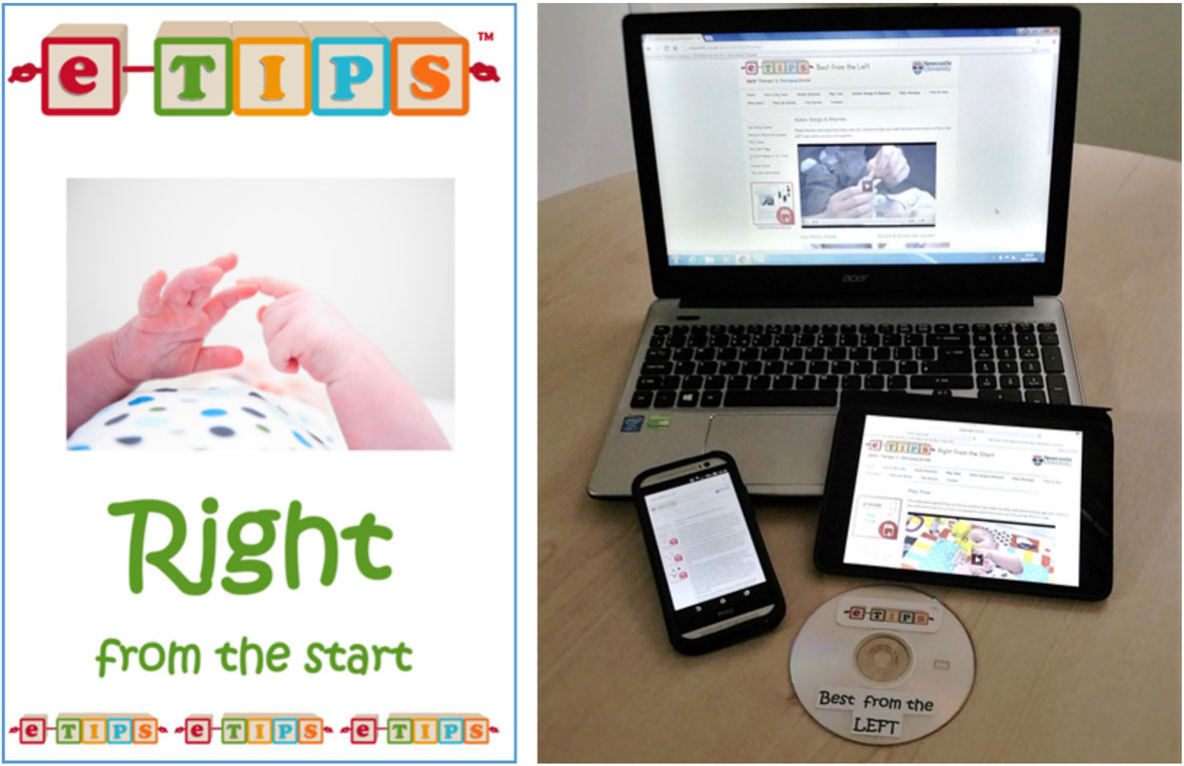
eTIPS materials. Left – cover of eTIPS manual “Right from the Start”. Right – all materials including videos available on website through PC, tablet or smartphone, and on DVD

Table 2 summarises the intervention as per the TIDIER checklist [77] and Fig. 5 illustrates the materials provided to families. The main ingredient of the intervention can be summarised as supporting parents to provide a pervasive, lateralised therapy approach to the infant in the first 6 months of life. Simple straplines for each approach (“Best from the Left”, and “Right from the Start”) are used to help reinforce the main message. The mode of delivery is through parental education (face to face and through a manual, website and videos) regarding the intervention and the underlying rationale. Support of the parent with the required behaviour change is through discussions during monthly home visits, with interim texts and phone calls, as well as the use of mind maps as preparation for engagement with eTIPS.

**Table 2:** eTIPS: Summary of the intervention, structured according to TIDIER checklist

BRIEF NAME	eTIPS – Early Therapy in Perinatal Stroke
Why	Early intervention to promote activity of the potentially affected side of the body in the first 6 months of life after perinatal stroke could improve motor outcome through activity-dependent plasticity.
What	Main ingredient: parental support with delivery of a pervasive, lateralized therapy approach in the first 6 months of life. The aim is to promote active movement, sensory input and visual stimulation to the potentially affected side of the body, but incorporate the approach into activities of daily life (feeding, playtime, changing, bath time, when out and about etc.)
Who provides	Currently the eTIPS team provide training and support to parents/carers, who deliver the eTIPS approach to infants. The training and support could be delegated (with development of a training resource) to a community physiotherapist or health visitor.
Modes of delivery of training to parents	Materials for participants – manual, website, DVDs, ribbon, stickers, diary, mini-guides, mind maps, checklists Materials for fidelity of training/education of parents – checklist and Perinatal Stroke information Materials for checking components delivered – checklist Materials for training intervention providers – to be developed in next phase Procedures: Initial visit with education and training. Principles reinforced through monthly follow-up visits and fortnightly phone calls until 6 months corrected age. Weekly texts provide prompts and an opportunity for troubleshooting.
Where	In the child’s environment – at home or out and about
When and how much?	1st 6 months of life (or for preterm infants, from term-equivalent age to 6 months age corrected); pervasive
Tailoring	Side of intervention is side of the body at increased risk of developing motor difficulties (hence 2 different manuals – Right from the Start, and Best from the Left)
Modifications	Modifications made following focus groups are summarized in Table 1
How well (Fidelity)	Planned: Interviews, Telephone calls; Home visits; Texts; Diaries Checklists to use when training parents

The approach is designed to be interactive and enjoyable. Through the intervention, parents promote active movement and provide sensory input and visual stimulation to the infant biased to the potentially affected side of the body. The approach exploits the use of minor modifications to the infant’s activities of daily living (feeding, playtime, changing, bath time, when out and about etc.) in order to maximise parental engagement and minimise any burden. The manual, videos and website provide highly pictorial, clear messages about how to do this, including suggestions for activities and play. Involvement of other family members including siblings is actively encouraged. Rather than focussing purely on sensorimotor function, the materials also model positive parenting behaviours and parent-infant bonding, providing guidance on how to interpret and respond to aspects of infant behaviour and avoid overstimulation. The manual provides guidance on how to modify the approach as the infant develops: this is intended to be supplemented by therapist support. The approach is currently being evaluated within a pilot feasibility study, and we are developing a training package for therapists to allow greater reach of implementation and therefore evaluation.

## Discussion

This is to our knowledge the only early therapy intervention aimed at the first 6 months of life after perinatal stroke. The rationale for this very early intervention in terms of a high level of central nervous system plasticity has been discussed above. Intervention in this time window brings particular challenges as discussed below, which make a participatory design approach and Normalization Process Theory framework [66] particularly important in developing an intervention which will be understood and accepted by stakeholders.

Behaviour change interventions are notoriously difficult to implement [78], even outside the context of sleep-deprived families coming to terms with the disruption of a new diagnosis in their infant child [79]. Furthermore, following a diagnosis of perinatal stroke, families experience a range of emotions including feelings of guilt and self-blame [74, 75]. There is also uncertainty about the future in terms of the nature and severity of difficulties their child may face. Some parents express feelings of helplessness, wishing there is something they can do. We were sensitive to the need to create materials and activities designed for parents in their capacity as parents during everyday interactions with their children, rather than requiring them to take on therapist roles during defined “therapy sessions”. This pervasive approach has a high potential for interactional workability, embedding the therapy into everyday routines and thus normalising the method, as well as avoiding conflict between perceived roles as parent versus therapist [62]. Supporting parents to deliver therapeutic input to their infants in this context can be seen as appropriate but challenging, and requires consideration for the welfare of both parties. Furthermore, engagement of such young infants in activities is highly dependent on positive parent/infant interactions, with parents providing the physical, interactional and emotional setting in which the infant is motivated to initiate the required behaviours. However, parents are in general highly motivated to improve outcomes for their infants, and our experience with the parent focus groups also revealed a wish to have been able to engage with a program like eTIPS. We deliberately made our materials as visually appealing as possible and framed most of the activities within the context of everyday parenting and play, allowing some tailoring to the specific requirements of individual families. In our ongoing pilot feasibility study we formally monitor parental wellbeing [80] and parenting sense of competence [81, 82].

The evolution of motor difficulties following perinatal stroke adds a further layer of complexity in explaining an early intervention approach to parents and indeed to healthcare professionals. Whilst early definitive neuroimaging is highly predictive of motor outcome after perinatal stroke [55–57], it is difficult for parents and therapists to understand why doctors may be worried about the future development of hemiparesis in an infant who may initially have no lateralised motor signs. This requires careful explanation and support.

A major strength of the eTIPS approach is the use of participatory design processes during intervention development, involving parents and healthcare professionals. Furthermore, the materials developed were considered straightforward, user-friendly and appealing. A challenge for monitoring intervention fidelity is the ecological nature of the approach (which in every other way we see as a strength) – it is designed to be pervasive and flexible around the child’s microenvironment rather than occurring in predefined windows of intensive input of a fixed nature. It is not possible to quantify the “dose” of an intervention delivered in this format. However, the basic message and strapline is straightforward and the explanations are clear. Manual contents can be used as a fidelity checklist, and correctness of the approach observed and discussed at home visits, or videoed by families. In our pilot feasibility study we use qualitative research methods including comprehensive observations and in-depth interviews to explore to what extent different aspects of the approach are found to be helpful, adopted and routinized by different families.

One weakness of the study is that focus groups were undertaken in the Northern region only. However, we did also obtain feedback from the London-based Child Stroke Research Reference Group, although this group is not specific to perinatal stroke. In addition, our local impression of the range of therapist practice in infants with perinatal stroke was confirmed through our national survey as well as through discussion with other therapists within Europe during meetings/workshops. It seems likely that the eTIPS approach will be valuable for infants with perinatal stroke in a number of contexts and settings, if they are diagnosed within the first few months of life. This brings up another issue – some of the parents in our focus groups had children with presumed perinatal stroke rather than symptomatic stroke. For these parents, diagnosis was delayed beyond the first few months of life. We elected to focus on a therapy approach for the first six months because of the current lack of a standardised therapy approach or resource devoted to this time window, as well as the potential to influence activity-dependent plasticity in the developing nervous system.

Parents of infants or very young children diagnosed with perinatal stroke were excluded from the focus groups, because we wanted to avoid undue distress to families in whom the diagnosis might still be evolving and in whom we were not in a position to offer a therapy package at that stage. Clearly these parents are the immediate stakeholders, and it is possible that some of their views might differ from those of parents with older children who had a perinatal stroke. We are currently undertaking a mixed methods pilot feasibility study of the eTIPS intervention, which will provide rich data on the views and feedback from these stakeholders.

The eTIPS approach has the potential for broad reach [83], given its low cost and availability of materials through the internet. There are options for further development of materials and perhaps for online peer support, which has been shown to increase the effectiveness of web-based interventions [84]. If found to be effective, adoption and implementation will have been facilitated by the participatory design and use of NPT, involving stakeholders throughout the intervention development process.

## Conclusions

A participatory design process and Normalisation Process Theory framework have helped us to develop a parent-delivered complex intervention, namely the early therapy in perinatal stroke (eTIPS) program. This program addresses a current gap in therapy intervention practice for infants with perinatal stroke during a period of high central nervous system plasticity. We are currently undertaking a pilot feasibility study of the eTIPS approach with a view to further evaluation within a randomised controlled trial.

## Abbreviations

eTIPS: early therapy in perinatal stroke
NIHR: National Institute of Health Research
NPT: normalisation process theory
